# Fish couple forecasting with feedback control to chase and capture moving prey

**DOI:** 10.1098/rspb.2024.1463

**Published:** 2024-09-25

**Authors:** Benjamin T. Martin, David Sparks, Andrew M. Hein, James C. Liao

**Affiliations:** ^1^ Department of Theoretical and Computational Ecology, University of Amsterdam, Science Park 904, Amsterdam 1098 XH, The Netherlands; ^2^ Department of Biology, The Whitney Laboratory for Marine Bioscience, University of Florida, Saint Augustine, FL 32080, USA; ^3^ Department of Computational Biology, Cornell University, Weill Hall, 102, Tower Rd, Ithaca, NY 14850, USA

**Keywords:** predator–prey, pursuit, feedback control, sensory-motor delay, forecasting

## Abstract

Predator**–**prey interactions are fundamental to ecological and evolutionary dynamics. Yet, predicting the outcome of such interactions—whether predators intercept prey or fail to do so—remains a challenge. An emerging hypothesis holds that interception trajectories of diverse predator species can be described by simple feedback control laws that map sensory inputs to motor outputs. This form of feedback control is widely used in engineered systems but suffers from degraded performance in the presence of processing delays such as those found in biological brains. We tested whether delay-uncompensated feedback control could explain predator pursuit manoeuvres using a novel experimental system to present hunting fish with virtual targets that manoeuvred in ways that push the limits of this type of control. We found that predator behaviour cannot be explained by delay-uncompensated feedback control, but is instead consistent with a pursuit algorithm that combines short-term forecasting of self-motion and prey motion with feedback control. This model predicts both predator interception trajectories and whether predators capture or fail to capture prey on a trial-by-trial basis. Our results demonstrate how animals can combine short-term forecasting with feedback control to generate robust flexible behaviours in the face of significant processing delays.

## 1. Introduction

Predator**–**prey interactions shape the evolutionary and ecological dynamics of biological communities [1–3]. Central to understanding the evolutionary and ecological consequences of predator–prey interactions is predicting when encounters between predators and their prey result in capture. Such outcomes depend not only on readily measurable locomotory traits like speed and manoeuvrability [4–6] but also on the intricate coupling of these traits with behaviour [7]. Advances in computer vision and animal tracking now enable detailed reconstruction of behaviours of individuals during interactions, allowing for the reverse engineering of behavioural rules that underlie ecological interactions [8–10]. Using these methods, a series of recent studies on fish [11], birds [12–14], flies [15,16], bats [17] and walking insects [18] have demonstrated that the trajectories of predators during pursuit can be predicted with remarkable fidelity by simple mathematical models that describe how predators steer in response to the perceived location and motion of their prey. A common finding among these studies is that the behaviour of predators is consistent with simple feedback control rules, whereby the predator turns at a rate proportional to low-dimensional sensory features that convey the relative location or motion of the target. By iteratively turning in this way over the course of the pursuit, the predator can appropriately couple its own motion to the motion of its target and generate a collision course [19]. If such simple rules underlie predator–prey interactions, this would be a powerful result for ecology because it suggests that tractable models of interception could be used to predict the outcomes of predator–prey interactions in a way that might be generalized across different kinds of ecological systems [20].

Crucially, understanding the specific behavioural rules predators use during interactions with prey is essential for inferring conditions that lead to successful captures. For instance, if predators rely on feedback control alone, then the inherent processing and transmission delays in biological brains—sensory-motor delays of tens to hundreds of milliseconds [20]—should have predictable consequences for interception performance. These delays mean that a predator’s actions are responses to outdated sensory information. As a consequence, strategies based on feedback control with delayed sensory inputs (hereafter referred to as delay-uncompensated feedback control) become highly unstable when facing rapid trajectory changes by their targets [21]. This instability can be manifested as a ‘loss of control’ in which the pursuer drastically overcorrects its trajectory in response to a target manoeuvre, causing the predator to miss its target [16,21]. Under delay-uncompensated feedback control, the risk of losing control can be mitigated only by lowering sensitivity to target motion, but this comes at the cost of being less responsive to target manoeuvres. So, if animals use delay-uncompensated control strategies to intercept prey, they should be forced to either risk losing control of their steering while pursuing manoeuvring targets, or reacting slowly to target manoeuvres, either scenario reducing the probability of capture.

Whether predators truly rely on delay-uncompensated feedback control to intercept prey is still uncertain; to date, no study has directly compared delay-uncompensated control against other hypothesized forms of motor control to explain the interception trajectories of predators. For instance, a large body of research in neuroscience suggests that humans and other animals cope with sensory-motor delays when generating lower-level motor behaviours such as eye movements [22,23], reaching [24] and balancing [25] by forecasting future sensory input, rather than relying on delayed input alone. Moreover, there is some evidence predators make use of forecasts when capturing evasive prey. For instance, during prey interception, dragonflies maintain the image of their target within a specialized high-acuity region of their eye (the fovea) by predictively rotating their heads to compensate for their own whole-body movements [26]. Furthermore, animals employing open-loop ballistic attacks, such as salamanders projecting their tongues to catch flies or archerfish spitting water jets to knock down terrestrial prey, aim their strikes at forecasted future target locations to account for the target’s movement during the sensory-motor delay [27,28]. Thus, forecasting appears to be involved in many tasks that require interactions with targets—an observation that contradicts assumptions of delay-uncompensated models of control.

Here, we set out to answer two open questions about the mechanisms by which predators intercept prey. Firstly, we sought to directly test whether animals perform delay-uncompensated feedback control or whether they combine feedback with some form of forecasting to intercept prey. Secondly, we sought to determine whether, in addition to predicting how predators move during interception, simple models of predator interception behaviour could accurately predict prey capture success—a fundamental ecological rate with broad relevance to predator–prey systems. Directly comparing models of delay-uncompensated feedback control to models of predictive control has been challenging, in part, because past studies have typically measured how pursuers move when intercepting targets that travel along smooth trajectories [11,12,14–16,18] (but see [13]). Under such conditions, linear motion extrapolation provides a good estimate of future target motion, but delayed sensory inputs do as well, making it challenging to discriminate between the two kinds of strategies. To address this, we used a novel naturalistic experimental system to present moving virtual prey to feeding fish (rainbow trout, *Oncorhynchus mykiss*) in a way that allowed us to generate discontinuous perturbations to target trajectories (‘target manoeuvres’) near the time of interception. Animals were consistently able to quickly adjust their trajectories to a new course that resulted in capture or near capture. Simple delay-uncompensated control strategies could not reproduce this behaviour and instead reacted either too slowly, or lost control completely following target manoeuvres. We introduce a simple, forecast-based control model that overcomes these issues by combining linear motion extrapolation with a separate forward model of self-motion. We show that this model predicts both successful adjustments and misses after target manoeuvres on a trial-by-trial basis. Our findings suggest that animals maintain rapid but robust control during interception by combining motion forecasting with feedforward prediction of self-motion using a simple computational control strategy that need not be precisely tuned to the motion of any given target.

## 2. Results

### (a) Interception performance cannot be explained by delay-uncompensated feedback control

**Figure 1.**
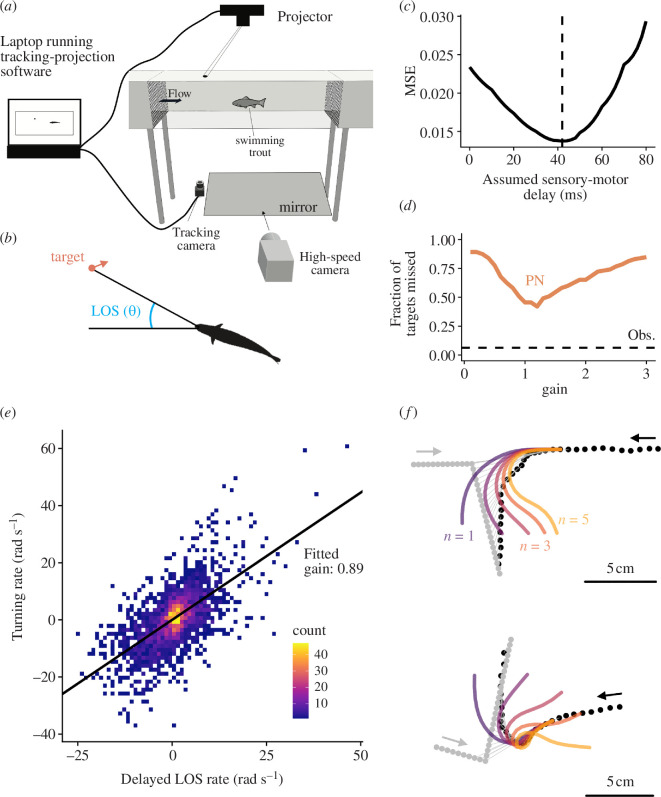
Interception performance and behaviour of fish in virtual feeding experiments cannot be adequately reproduced by delay-uncompensated feedback control. (*a*) Diagram representing the setup in which virtual moving targets were projected above feeding fish swimming in a flow tank. (*b*) The line of sight (LOS) to the prey is defined as the angle to the target from the fish’s head with respect to an internal reference frame. In proportional navigation (PN), turning is proportional to the rate of change of LOS which is caused by movement of both target and fish. (*c*) Sensory-motor delay was estimated using a change-point detection method applied to the fish’s velocity vector following a change in the target trajectory (see §4). Panel shows the mean square error (MSE) of the velocity vector across all trials (*n* = 112) as a function of the assumed sensory-motor delay. (*d*) Comparison of interception performance (measured as the fraction of trials in which modelled trajectories reached within a predefined capture distance) between PN models with varying gains, and observed interception performance in experiments. (*e*) The observed relationship between turning rate and delayed LOS rate in the experimental trials. (*f*) Two examples of fish interception trajectories (black points) against manoeuvring virtual targets (grey points), showing that simulations of PN (coloured curves) fail to accurately represent the trajectories of fish irrespective of the assumed gain. Lower gains, *n*, lead to under responsive modelled interceptions, while higher gains cause unstable, overcompensating turns. Grey and black arrows represent the direction of movement of the target and fish respectively. Points are separated by 10 ms intervals.

We conducted experiments with *O. mykiss* in a flow tank (figure 1*a*), which allowed us to recreate in the laboratory, the type of conditions under which animals feed in nature [29]. For each experimental trial, we projected a moving virtual target (small dark circle) onto a semi-transparent projection screen on the surface of the water. Fish in the experimental trials readily attacked and attempted to ingest the virtual targets as evidenced by a rapid expansion of the buccal cavity at the terminal stage of interception manoeuvres (electronic supplementary material, video). Targets moved in straight trajectories except during manoeuvre events, where the target changed trajectories to a new heading. By programming target manoeuvres to occur within a close range of a station-holding fish and varying the speed of the target, we generated trajectories that would induce a strong trade-off between sensitivity and stability for a delay-uncompensated controller [18,20,21], where sufficiently high turning gains are required to respond to the target manoeuvre, but at the same time, such gains are prone to generate unstable, overcompensating turns.

Despite its inherent difficulty, animals excelled at this interception task, coming within 1.5 cm of the target on ~92% of trials (*n* = 112). The distance, 1.5 cm, represents a critical distance threshold for these animals when feeding on real prey. Beyond this distance, strikes are more likely to miss than to capture prey (electronic supplementary material, figure S1).

A strength of the literature on animal pursuit behaviours is that hypotheses about how an animal takes in sensory data and transforms those data into movement decisions are typically articulated as mathematical models that predict behaviour as a function of sensory input. These models specify which sensory data are used, and—at an algorithmic level of description [7]—how the brain operates on those data to drive manoeuvres [12,16]. This formalism allows one to make precise, quantitative predictions about behavioural output, and to compare those predictions to observations. Using this approach, we compared the performance of animals during experiments to the modelled performance of animals that use a simple, widely invoked feedback control rule known as ‘proportional navigation’, PN [12,16,19]. PN takes in delayed visual input (the rate of change of the line of sight to the target, line of sight (LOS) rate, see figure 1*b*) and produces steering manoeuvres as output using a single ‘turning gain’ parameter to control sensitivity (see §4). For each empirical interception trajectory, we used the observed initial conditions of the fish and target, then simulated an interception trajectory assuming the animal controlled its steering using PN with an empirically determined sensory-motor delay (42 ms; figure 1*c*). The turning gain that resulted in the highest interception performance for PN was ~1 (figure 1*d*). Moreover, the slope of the relationship between the instantaneous turning rate of fish and the delayed LOS rate indicated that a gain slightly below 1 (0.89; 95% CI: 0.85–0.93) was most consistent with data (figure 1*e*). However, irrespective of the assumed turning gain, simulated interception trajectories performed significantly worse than real fish at intercepting targets, (figure 1*d*).

We next sought to determine whether the poor performance of PN was caused by a trade-off between responsiveness and risk of losing control during interception manoeuvres as predicted by past theoretical work [18,20,21]. In agreement with this, we found that the trajectories predicted by PN with gain in the fitted range were underresponsive compared with the observed interception trajectories of animals following a target manoeuvre (figure 1*f*; purple trajectories). Increasing gain to rectify this initial underresponsiveness resulted in severe overcompensating turns later in the trajectory (figure 1*f*; orange and yellow trajectories). Thus, evidence from both the behavioural (interception trajectories) and performance data (miss frequency) suggest that neither PN nor other delay-uncompensated control rules (electronic supplementary material, figure S2) could account for observed interception behaviour.

### (b) Responsiveness to LOS rate is not constant but changes predictably with time

**Figure 2.**
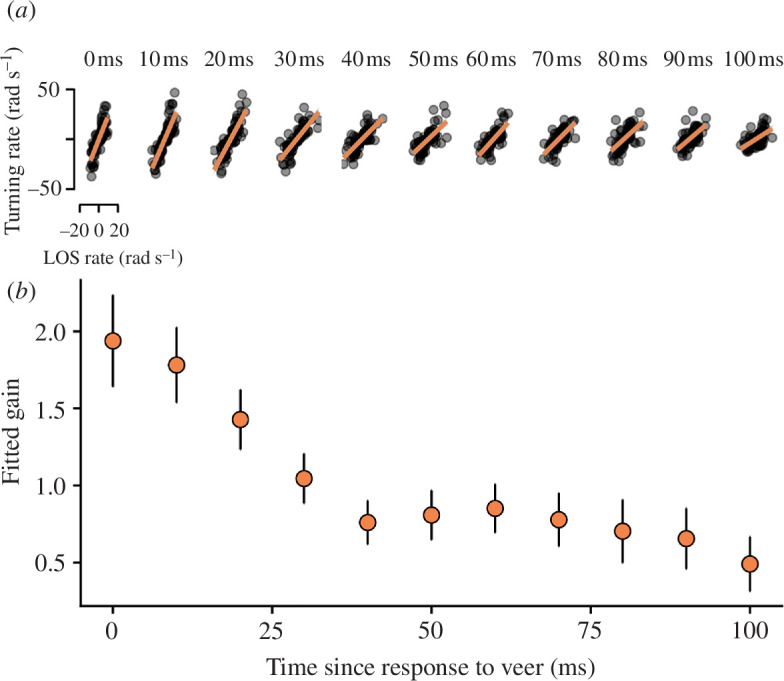
Systematic variation in the relationship between turning rate and delayed LOS rate over time. (*a*) The observed relationship between turning rate and LOS rate for each interval of 10 ms following the initial response to the target manoeuvre (40 ms after the manoeuvre). (*b*) The slope characterizing the relationship between turning rate and LOS rate demonstrates a systematic decrease over time. Points are estimated slopes for each time interval during the first 100 ms following the initial response to the manoeuvre in single manoeuvre trials; bars are 95% CIs.

One way to understand how observed manoeuvres deviate from predictions is to fit PN sequentially to short segments of trajectory following the perturbation and to estimate the gain for each segment. We found that a systematic decrease in gain over the course of a manoeuvre is required to match data (figure 2), where the gain must be high immediately after the target manoeuvre to match rapid responses by animals, and decrease progressively over time to match the stability observed in the later stages of interception (figure 1*e*). This analysis reveals why constant gain PN cannot explain the data: animals do not respond in a consistent way to the LOS rate over the course of interception.

We hypothesized that the apparent time-varying gain may not be an interception rule itself, but rather the consequence of predictive control. More specifically, the high sensitivity of turning immediately following the perception of the manoeuvre is suggestive of predictive control because even though the target has only moved a small distance, the expected future position of the target has changed substantially. To explore this hypothesis, we compared interception trajectories to the predictions of two alternative models of predictive control.

### (c) Testing models of predictive control

A motivating logic behind proportional navigation is that, under this strategy, one can achieve interception *without* the need to separate target motion from self-motion. All that is important is relative motion, meaning that only a single variable—change in LOS rate—needs to be encoded in the brain. We therefore first sought to test whether a simple extension of PN based only on relative measures of motion could explain the interception performance of animals. We did so with an extension of PN, which we refer to as predictive PN, where rather than turning in response to the delayed LOS rate, turning is proportional to the expected current LOS rate based on a linear extrapolation of this variable up to the present time. In principle, this strategy could overcome sensory-motor delays at a low-computational cost because only LOS rate needs to be encoded and forecasted.

**Figure 3.**
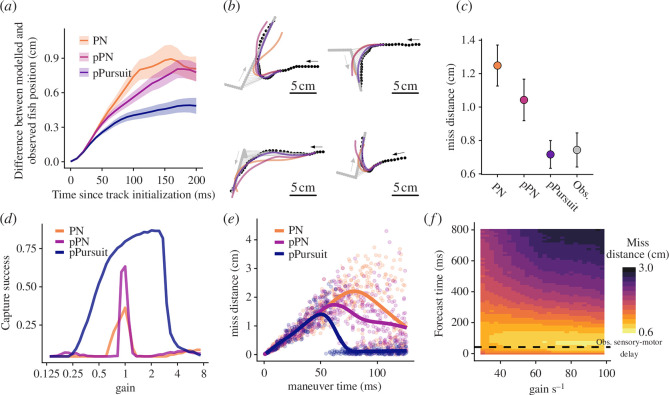
Comparing delay-uncompensated and predictive models of guidance control. (*a*) Comparison of different guidance rules’ abilities to predict the position of fish as a function of time since model initialization. Lines denote mean error across all trials for a given model run-time, shaded regions denote 95% CI across 112 trials. PN, proportional navigation; pPN, predictive PN; pPursuit, predictive pursuit. (*b*) Four trajectories of fish (black points) pursuing targets (grey) with modelled trajectories for the different guidance rules. Grey and black arrows represent the direction of movement of the target and fish, respectively. Points are separated by 10 ms intervals. (*c*) Performance of the different guidance rules at intercepting the virtual targets in experimental trials (measured by distance to the target at the end of the trajectory) compared with the observed interception performance of fish in the experiments. Both PN and pPN have average miss distances significantly higher than the observed miss distance (PN: *p* = 1.2 × 10^−8^; pPN: *p* = 0.0012), while the miss distances predicted by pPursuit were not statistically different than observed miss distances (*p* = 0.45). Models were implemented with gains that minimized the difference between observed and predicted trajectories (e.g. panel (*a*)). (*d*) Capture success (see §4) in simulated interception trials for the three guidance rules as a function of relative gain (a multiple of the fitted gain from the experimental trials for each model). (*e*) Miss distance as a function of the manoeuvre time, where manoeuvre time is defined as the time-to-collision at time of target manoeuvre. (*f*) Interception performance of predictive pursuit in simulated target interceptions as a function of forecast time horizon and gain. Interception performance is maximized when forecasts are approximately equal to the duration of the sensory-motor delay (42 ms).

The extension of PN to include a forecast of current LOS rate improved agreement between model predictions and observed trajectories (figure 3*a*). However, predictive PN still produced trajectories that were underresponsive in some cases and overcorrecting in others (figure 3*b*) relative to observed interception manoeuvres. Moreover, predictive PN was not capable of matching the observed interception performance of animals in our experiments (figure 3*c*). The predictive PN model represents a special case of a proportional-derivative (PD) controller, where turning is driven by a linear combination of both the LOS rate and its time derivative. In predictive PN, the gain for the derivative term is equal to the gain of the proportional term times the sensory-motor delay (see electronic supplementary material, figure S3). We also tested whether a PD controller with two free gain parameters could better explain the predators’ trajectories, however, this too only led to modest improvements in model performance (see electronic supplementary material, figure S3).

A potential weakness of predictive PN is that LOS rate convolves both target motion and self-motion into a single control variable; but, target motion and self-motion are *not equally difficult to predict*. Indeed, in vertebrates including fishes, the cerebellum is capable of learning and encoding internal models (sometimes referred to as ‘forward models’) that predict the sensory consequences of self-motion [30–32]. Such models are thought to be involved in calibrating the intensity of a motor response to achieve a desired motion [32]. We propose this same neural mechanism could allow animals to anticipate the consequences of their own recent motor commands on their relative position and orientation with respect to the target, and thus allow animals to isolate absolute measures of target motion from visual features such as LOS rate that encode only relative motion.

Based on this hypothesis, we propose a predictive guidance rule that extends the delay-uncompensated proportional pursuit guidance rule [16,18,19]. In proportional pursuit, turning is proportional to the error angle, defined as the difference between the heading of the predator and the LOS to the prey: $\frac{dh}{dt}=k\left(\theta \left(t-\tau \right)-h\left(t-\tau \right)\right)$ , where  is the heading of the fish, θ is the line of sight to the target, and τ is the sensory-motor delay, such that $\theta \left(t-\tau \right)$ represents the line of sight at a period of one sensory motor before the current time. In a guidance rule, we refer to as ‘predictive pursuit’, we postulate that the animal predicts the current error angle at time *t* rather than operating on delayed sensory inputs, and does so using extrapolation of target motion to predict target position at time *t* [28,33], and a forward model of self-motion to accurately infer its current position and orientation at time *t* [25,26,31]. Our model thus assumes that steering relies on separate encodings of target motion [28,33] and self-motion [25,26,32], each of which is then forecast separately, and combined to compute the current error angle. We postulate that this segregation is made possible by the same cerebellar circuits that are involved in self-motion prediction and correction during optomotor responses [31,32,34].

Comparing predictions of the predictive pursuit model to data showed that it outperformed both delay-uncompensated and predictive PN in explaining observed patterns (figure 3 *a,b*). Importantly, model simulations employing predictive pursuit, much like the observed trajectories of fish in the experiment, were highly responsive to rapid changes in the target trajectory following a target manoeuvre, but avoided overcompensating turns in the final stages of interception (figure 3*b*, dark purple curves). Predictive pursuit was also the only guidance rule capable of matching the observed interception performance of fish (figure 3*c*), and was furthermore able to explain variation in capture success on a trial-by-trial basis (electronic supplementary material, figure S4).

We next sought to understand which features of predictive pursuit were responsible for its improved performance compared to PN and predictive PN. To do this, we conducted simulations of the proposed guidance rules across a range of target trajectories, where we varied the speed, timing and direction of simulated manoeuvres. Across all manoeuvre parameters, predictive pursuit outperformed both PN and predictive PN for the fitted gains from the experimental trials (figure 3*d*). Moreover, unlike PN or predictive PN, predictive pursuit exhibited robust interception performance over a broad range of gains (figure 3*d*), indicating that by separately forecasting target and self-motion and using these to drive steering, an individual need not precisely tune its gain to the motion kinematics of a target.

Interestingly, there were also systematic differences in the types of manoeuvres that were most effective against the different guidance rules. Against PN and predictive PN, manoeuvres were most effective if initiated when the time to collision with the predator was approximately twice the sensory-motor delay of the predator, allowing prey time to move sufficiently far from its original heading and in turn generate larger miss distances (figure 3*e*). However, against predictive pursuit, such early manoeuvres allowed sufficient time for predators to reestablish a collision course, and intercept the prey with small miss distances. Thus, against predators implementing predictive pursuit, prey had to initiate evasive manoeuvres much later (~1 sensory-motor delay until collision), resulting in substantially lower miss distances.

If forecasting the present state of the world improves interception performance, a natural question is whether performance could be further improved by forecasting further into the future. For example, Yoo *et al.* [35,36] found evidence that macaques playing a joy-stick-controlled pursuit game steer toward the predicted location of targets at a point in the future (~800 ms). We tested whether animals would benefit from such long-term forecasts by running simulations across a range of forecast time horizons. Results show that forecasting the location of the target over time intervals substantially longer than the sensory-motor delay actually degraded interception performance (figure 3*f*). This is because, during the terminal phase of interception as the time to collision with its target approaches zero, steering towards the expected future target location can cause the predator to turn off of a collision course, and thus miss its target.

## 3. Discussion

At the scale of ecological populations, predator–prey dynamics unfold as the result of vast numbers of interactions between individual predator–prey pairs. The rate of these interactions depends not only on a predator’s ability to encounter prey [37] but also on their ability to capture the prey they encounter. While past work has strongly emphasized biomechanical constraints on predator–prey manoeuvres [4,38], the behavioural algorithms predators use to decide how to move during interactions with prey are at least as important [20]. Here, we have combined algorithmic models with a novel experimental system to reveal the core computational rules by which predators intercept evasive, fast-moving prey. Our results reveal that two key principles that have previously been described in the context of human motor control—responses to visual feedback [39,40], and forecasts of how self-motion will affect future feedback [23]—are also central to the strategies predators use to intercept fast-moving prey. Our findings also build on past studies of target interception in animals [12–16,26,33], by revealing that (i) simple, tractable models of pursuit behaviour are capable of predicting both predator manoeuvres and capture success, and (ii) observed predator interception behaviour cannot be explained by delay-uncompensated feedback control alone [12–15] or by simple motion extrapolation alone [28]; rather, predators drive interception manoeuvres by predicting the *future feedback* they will receive as both they and their targets move.

Recent literature proposes that the interception behaviour of predators can be explained by delay-uncompensated control [12–16], whereas many lower-level behaviours involving motor control of eye, head and hand movements appear to involve short-term forecasting of current or future states of the world [23,26,41]. Our results help resolve this apparent conflict by studying interception behaviour in a regime where delay-uncompensated control alone becomes unstable. We show that predators pursue moving prey by combining feedback control with elements of forecasting. Our results further demonstrate that the nature of the predictive model employed by the brain matters a great deal. A simple extension of feedback control that linearly forecasts the expected sensory input—conflating self and target motion—resulted in only a marginal improvement in responsiveness. In contrast, we find that two simple behavioural modules of forecasting—a feedforward model of self-motion [31,32] and extrapolation of target motion [28,33]—can be combined with feedback control to generate the robust interception behaviours we observed in experimental animals.

Our results may help shed light on the critical ecological problem of predicting how capture success contributes to variation in interaction strengths between predators and their prey. Capture success varies drastically among different kinds of predator–prey pairs [42], yet ecology lacks general theory to predict how traits of both predator and prey contribute to the outcome of interactions. Biomechanical performance parameters such as maximum acceleration and manoeuvrability vary predictably with key organismal traits such as body size and mode of locomotion [4–6], suggesting the possibility that capture success might be predictable from first principles across very different types of predator–prey interactions. Yet, without an understanding of how predators use their biomechanical capabilities to construct interception manoeuvres, it is impossible to translate such relationships into predictions about capture success [20]. Our results indicate that simple, tractable models of pursuit behaviour can predict both average capture success and variability in capture success across encounters due to the timing and speed of prey manoeuvres. This suggests that despite the apparent complexity of these behaviours, a mechanistic and predictive theory of predator–prey engagements may be possible. Such a theory could help resolve why capture probability varies so drastically among different kinds of predators and prey, and to understand how variation in species traits and behaviours determine the rate at which encounters result in capture.

Our results also reveal a strong connection between a crucial ecological behaviour, intercepting moving prey, and classic questions about the neural basis of sensory-motor control, thereby linking ecology to neuroscience. In particular, our results were most consistent with the hypothesis that to achieve high interception success, predators encode separate representations of target motion and self-motion and use these to produce separate forecasts. In vertebrates, forecasts of self-motion are believed to be performed by what are known as ‘internal forward models,’ which are learned and encoded in the cerebellum [31,32,35]. In zebrafish, for example such models are capable of encoding the relationship between motor output and whole-field visual feedback resulting from self-motion, and these models can be updated through experience [32]. Within this context, internal models help identify the level of motor activity needed to reach a desired state even before visual feedback is received. We propose that these same internal models may also provide the neural basis for robustly intercepting moving targets. Specifically, our results suggest that animals use internal models for two key subcomponents of predictive pursuit: predicting the sensory consequences of self-motion during the period of sensory-motor delay, and subtracting out self-motion from relative motion to estimate target motion. For the first component, we propose that the internal model used for anticipating the consequences of self-motion on whole-field visual reafference would also allow animals to anticipate how their own actions during the sensory-motor delay period would affect their relative position and orientation with respect to a target. We further propose that animals then implement feedback control based on these anticipated, but not yet perceived, states. Secondly, while existing models of feedback control rely on visual features encoding relative motion, predictive pursuit requires an ability to directly estimate target motion. The discrepancy between the expected target motion due to self-motion alone and the target motion actually observed provides the key elements for estimating target velocity. It has long been known that the brain is capable of accurately estimating absolute target motion even when the observer is moving [43]. However, the reason for this ability has remained unclear. Our analysis demonstrates the functional significance of encoding both self-motion and target motion separately: doing so allows for more robust forms of predictive control compared with those relying solely on features of relative motion.

Generating flexible, robust behaviour in novel situations is a fundamental challenge all animals face. The problem of intercepting manoeuvring prey is emblematic of this challenge: a predator must tailor its behaviour to the dynamic trajectory of each prey it pursues. Yet, prey trajectories can vary wildly from one encounter to the next [44,45]. Feedback control offers a solution to this problem. By using dynamic sensory input from the prey to drive trajectory control, a predator can produce trajectories that are matched to the prey’s motion, whatever it may be. However, sensory-motor delays result in a trade-off between responsiveness and stability for delay-uncompensated control. Given the high-speed nature of predator–prey interactions, and the rapid, unpredictable nature of prey evasive manoeuvres [20,44,46,47], trade-offs between responsiveness and stability place fundamental limits on delay-uncompensated control. Predictive control can relax this trade-off, eliminating the need to precisely tune the parameters that relate sensory inputs to motor outputs. We thus expect this more robust form of control may drive behaviour in many predator–prey systems and in rapid sensory-motor tasks more generally.

## 4. Methods

### (a) Experimental fish

Thirty *O. mykiss* (4.5–9.5 cm total length) were transported from Wolf Creek National Fish Hatchery, Kentucky, USA to the Whitney Laboratory for Marine Bioscience, Florida, USA. Fish were reared in a 180 l tank and fed a low ration diet (~0.25–0.5% of body weight per day) of crushed fish pellets (Purina Aquamax Sportfish MVP, St Louis, Missouri, USA). Water temperature was maintained at 15 ± 1°C using an inline chiller (Arctica Titanium Chiller, JBJ Business Center, Inglewood, California, USA). A divider was placed in the rearing tank upon the start of experimentation so that, after individual fish were used for experimental trials, they were released to the other side of the divider. After all fish from the first side were used once in experimental trials, the divider was removed and fish were used again for experimental trials.

### (b) Experimental system

Experiments were conducted in a 175 l recirculating flow tank (30 cm height × 25 cm width × 89 cm length; Loligo Systems, Tjele, Denmark) and maintained at 15 ± 1°C with an inline chiller (Delta Star chiller, model DS−4-TXV, Aqua Logic, San Diego, California, USA). A short throw projector (1920 × 1200 resolution, 60 Hz refresh rate; Epson BrightLink 696Ui, Epson America, Los Alamitos, California, USA) was fixed over the working section of the tank to cast a rear projection onto a lexan sheet, which in turn was secured over the top opening of the working area and painted with Goo rear projection coating (Goo Systems, Kingston, Ontario, Canada). A high-speed camera (100 fps, 2560 × 1600 pixel resolution; LAB340, Vision Research, Wayne, New Jersey, USA) was positioned at a 45° angled mirror to capture the ventral perspective of the working area. Simultaneously, a USB camera (ZED 2, Stereo labs, San Francisco, California, USA) was placed underneath the working section of the tank and used in a closed loop algorithm to determine at what point stimuli should change directions based on fish head location (see §4c figure 1*a*). Fish naturally held station next to a rock (~10 cm diameter) placed in the rear portion of the flow tank, which encouraged site fidelity across trials.

### (c) Experimental protocol

For each trial, an opportunistically selected fish was moved from the rearing tank to the experimental flow tank. Fish were allowed to acclimate until either at least 5 min had passed or until the fish rose from the bottom of the tank (typically by holding position around a cylinder or rock). Following the acclimation period, virtual target trajectories were presented to the experimental fish. New experimental trials were presented approximately every 60–120 s for a readily responding fish. The number of trials conducted with a fish in a particular session varied among fish due to differences in the propensity for attacking the virtual stimulus. Sessions with experimental fish were terminated after fish repeatedly failed to respond to the stimulus (at least 10 trials). In some cases, fish did not respond to the stimulus in any trial, and thus in this case generated no usable data. After the experimental trials were completed for a fish, its length was measured, and it was put back into a separate division of the holding tank. Fish were then reused in experiments on subsequent days. To minimize handling and stress we left post-experimental fish unmarked. Because individuals were not labelled, we do not know the exact number of unique fish that attacked the target. However, based on differences in size (grouping fish into 0.5 cm length bins and assuming all fish within a bin are the same fish), a minimum of seven fish attacked the target with the number of attacks per size bin ranging from a minimum of 1 to a maximum of 34. This estimate of the number of experimental fish used is highly conservative, as lengths were measured to a precision of 0.1 cm and experiments were conducted over a three week period where growth was minimal.

Targets either manoeuvred once (single manoeuvre trials), or twice (double manoeuvre trials). In double manoeuvre trials, the second manoeuvre occurred 50 ms after the first manoeuvre and was directed in the opposite direction of the first manoeuvre, generating a zig-zag trajectory (electronic supplementary material, figure S5). The location of the first manoeuvre was controlled through a semi-closed loop algorithm, where the USB-camera was used to track the real-time location of the fish in the tank. The tracked position of the fish was used to determine the downstream (*x*) location of a target manoeuvre, with the location of the manoeuvre programmed to occur 5 cm upstream of the location of the fish. Motion of the fish over the interval between the initiation of the stimulus and the time of the manoeuvre resulted in a distribution of relative displacements between the fish and the target at the time of the manoeuvre ranging from 2 to 12 cm. The ground speed of the projected target was varied between 22 and 106 cm s^−1^. The manoeuvre angle, defined as the difference in the target trajectory before and after the manoeuvre, varied between 26 and 45 degrees.

Fish displayed no evidence of injury throughout the experiment, nor did they subsequently develop infections following the experiments.

### (d) Data processing

We included all experimental trials for which: (i) the fish initiated the pursuit before the first target manoeuvre, (ii) the position of the fish could be tracked through the entire trajectory (in a few trials the fish’s position was occluded by the rock placed to encourage site fidelity) and (iii) the fish attempted to capture the virtual target (indicated by a suction feeding attempt). This last criterion was used to exclude cases where fish initially turned or moved toward the target when the target was still at a distance, but then ceased this behaviour and did not actually attempt to intercept the target (see electronic supplementary material, video). Including data from such trials would risk conflating two different kinds of behaviour: inspecting a target (excluded from analysis) and attempting to intercept a target (included).

The two-dimensional position of the fish’s head was tracked using a custom Matlab script. The tracks were then manually inspected and corrected using a custom track-fixing GUI. The position of the virtual target was tracked manually with three keyframe positions, one when the virtual target first entered the working chamber of the tank, one frame at the time of the manoeuvre and one frame at the end of the trial. The position of the target at all other times was interpolated from these three time-points. Positions in pixel coordinates were converted to cm using a calibration image taken on the day of each trial.

For analysis, positions were converted to a flow reference frame by subtracting the flow velocity of the flume used in a trial multiplied by the amount of time passed since the initiation of the trial. The heading of fish was defined as the angle of the velocity vector. The turning rate of fish (figure 1*d*; figure 2) was defined as the time derivative of the velocity vector and was estimated using a Savitzky–Golay filter, with polynomial order 1 and a filter length of five steps.

### (e) Estimation of sensory-motor delay

We estimated the visual sensory-motor delay for fish by estimating the time delay between the target manoeuvre and the initiation of the fish’s response to the manoeuvre. To do so, we fit a piecewise linear model of the velocity vector angle as a function of time over a 100 ms interval following a target manoeuvre across all single-manoeuvre trials. We then evaluated the MSE of the piecewise model with breakpoint locations ranging from 0 to 70 ms after the manoeuvre. The sensory-motor delay was estimated by finding the breakpoint location which minimized the MSE of the piecewise model average across all single manoeuvre trials.

### (f) Interception models

We modelled the interception dynamics of different guidance rules, whereby the turning rate of the predator depends on either delayed (PN) or predicted sensory inputs (predictive PN and predictive pursuit). For PN, the turning rate is proportional to the delayed LOS rate


$$\frac{dh}{dt}=N\overset{˙}{\theta }\left(t-\tau \right),$$

where *N* is the navigation constant or gain parameter that relates turning rate to LOS rate ( $\dot{\theta }$ ), and τ is the sensory-motor delay. Predictive PN is similar to PN, except rather than turning in response to the delayed LOS rate, turning is proportional to the predicted LOS rate, which is estimated by a linear extrapolation of the delayed LOS rate:


$$\hat{\dot{\theta }}\left(t\right)=\dot{\theta }\left(t-\tau \right)+\tau \ddot{\theta }\left(t-\tau \right)$$

Finally, for predictive pursuit, turning is proportional to the angular difference between the predicted current LOS to the target and the current heading of the predator. We assume animals predict the current LOS through motion extrapolation to estimate the target’s relative position at time t , from its velocity at time t-τ . Furthermore, we assume that animals can estimate how their own motion contributes to a change in the relative position of the prey through an efference copy of motor commands over the interval [ t-τ , t ] and a forward model of self-motion. Consequently, the predicted relative position, or the range vector to the target at time t , is expressed as:


$$\hat{r}\left(t\right)=r\left(t-\tau \right)+\tau {\dot{r}}_{target}\left(t-\tau \right)+\int_{t-\tau }^{t}{\dot{r}}_{fish}\left(t^{`}\right)dt^{`}$$

where ${\dot{r}}_{target}$ and ${\dot{r}}_{fish}$ are the velocity vectors of the target and fish respectively. The angle of the predicted range vector, , along with the current heading of the fish determines turning rate:


$$\frac{dh}{dt}=k\left(\hat{\theta }\left(t\right)-h\left(t\right)\right)$$

For all models, we assume fish update their sensory inputs, and consequently, their turning rate at a frequency of 60 Hz, which is approximately the flicker-fusion frequency for *O. mykiss* in high light conditions [48]. The flicker-fusion frequency has been used in past analyses as an approximation of the effective update rate of sensory perception [17].

The equations of motion were integrated by the Euler method with a step size of 1.5 × 10^−4^ s, which we found was small enough to ensure negligible numerical error on modelled trajectories.

### (g) Model evaluation

To compare the predictions of the model to observed interception behaviour in the experimental trials, we initiated model simulations with the location and heading of the fish as initial conditions. For each time step, turning was governed by one of the guidance rules, and the speed of the model fish was set to the observed speed of the fish during that period. To evaluate the models’ abilities to predict the trajectories of fish, we initiated the model at every frame starting from the time of the manoeuvre until the frame before capture. We then recorded the average model error (the distance between the modelled location and the fish’s head) as a function of time since the model was initialized for each fish.

We estimated the best-fitting gains for each guidance rule by running each model with a range of gains, and finding the gain value that minimized the mean model error averaged across model initialization times from 0 to 200 ms. To avoid the risk of overfitting models to the data, we treated gain as a global parameter, where a single gain value was used across all experimental trials to model fish movement during interception. This effectively assumes that differences in gain from one trial to the next, and among individual animals, are small relative to other sources of variation in behaviour. Thus for each guidance rule, with the exception of the PD implementation of PN with two fitted gains, there was one fitted parameter for the entire dataset.

Once the best fitting gain was calculated for each guidance rule, we calculated both mean error and 95% CIs for model error across trials at different times since model initiation from 10 to 200 ms in 10 ms intervals (figure 3*a*). To compare model interception performance (e.g. capture success), we initialized models at the time of the target manoeuvre and integrated the equations of motion up to the time the experimental fish initiated a strike. Miss distance was defined as the minimum distance between the target and modelled fish position during the interception.

Our primary statistical analysis assumes that data from each trial are independent. However, to account for possible correlations in behaviour of separate trials performed with the same individual animal, we repeated the analyses treating individual fish identity as a random effect (see electronic supplementary material), where unique fish identities were assigned by differences in measured sizes of fish used in the trials (see §4c). Random effects models are a widely used statistical method for capturing complex correlation structures within data [49], such as that potentially introduced by including multiple trials from the same animal. None of the qualitative results presented in the main text were affected by including this additional correlation structure.

### (h) Simulations

We evaluated the performance of the various guidance rules at intercepting manoeuvring targets via simulations. In each simulation, the predator was initiated at a set distance from the target and moved at a fixed speed toward the target. The prey initiated an evasive manoeuvre when the time to collision with the predator reached a threshold value (hereafter, referred to as the manoeuvre time). Upon initiation of the manoeuvre, the prey moved at a fixed speed and angle, referred to as the manoeuvre angle, where a manoeuvre angle of 0 indicates the prey moved directly toward the oncoming predator. To explore the robustness of interception rules to the value of the gain parameter, we ran simulations across a range of gains for each guidance rule (from one-eighth to 8 times the fitted gain for each guidance rule). For each gain value for a particular guidance rule, we ran 1000 replicate simulations across a range of prey escape trajectories, by drawing the manoeuvre time (1–3 times the sensory-motor delay of the predator [42 ms]), manoeuvre angle (from  to ), predator speed (90–120 cm s^−1^), prey speed (0.6–0.8 times predator speed) from uniform distributions. The ranges of manoeuvre times and angles were selected so as to bound the optimal ranges of these parameters against predators implementing delay-uncompensated feedback control [20].

To explore how the performance of each guidance rule depends on the timing of the prey manoeuvre, we ran additional simulations where we varied the manoeuvre time from 0 to 3 sensory-motor delays. Manoeuvre angle, predator speed and prey speed were all drawn from the same uniform distributions reported above. For each guidance rule, we drew gains from a uniform distribution where the upper and lower bounds were set to 20% below and 20% above the optimal fitted gain from the experiments (1.15 for PN, 0.8 for predictive PN, 43.0 s^−1^ for predictive pursuit).

## Data Availability

Code and data required to reproduce the analyses of the study are available at [50]. Supplementary material is available online [51].
